# Theaflavin protects against oxalate calcium-induced kidney oxidative stress injury via upregulation of SIRT1

**DOI:** 10.7150/ijbs.57160

**Published:** 2021-03-02

**Authors:** Tao Ye, Xiaoqi Yang, Haoran Liu, Peng Lv, Hongyan Lu, Kehua Jiang, Ejun Peng, Zhangqun Ye, Zhiqiang Chen, Kun Tang

**Affiliations:** 1 Department of Urology, Tongji Hospital, Tongji Medical College, Huazhong University of Science and Technology, Wuhan, China.; 2 Department of Urology, The Second Affiliated Hospital of Kunming Medical University, Kunming, China.; 3 Department of Urology, The Third Affiliated Hospital of Chongqing Medical University, Chongqing, China.; 4 Department of Urology, Guizhou Provincial People's Hospital, Guiyang, China.

**Keywords:** Theaflavin, miR-128-3p, SIRT1, oxidative stress, nephrocalcinosis

## Abstract

Renal tubular cell injury induced by calcium oxalate (CaOx) is a critical initial stage of kidney stone formation. Theaflavin (TF) has been known for its strong antioxidative capacity; however, the effect and molecular mechanism of TF against oxidative stress and injury caused by CaOx crystal exposure in kidneys remains unknown. To explore the potential function of TF on renal crystal deposition and its underlying mechanisms, experiments were conducted using a CaOx nephrocalcinosis mouse model established by glyoxylate intraperitoneal injection, and HK-2 cells were subjected to calcium oxalate monohydrate (COM) crystals, with or without the treatment of TF. We discovered that TF treatment remarkably protected against CaOx-induced kidney oxidative stress injury and reduced crystal deposition. Additionally, miR-128-3p expression was decreased and negatively correlated with SIRT1 level in mouse CaOx nephrocalcinosis model following TF treatment. Moreover, TF suppressed miR-128-3p expression and further abolished its inhibition on SIRT1 to attenuate oxidative stress *in vitro*. Mechanistically, TF interacted with miR-128-3p and suppressed its expression. In addition, miR-128-3p inhibited SIRT1 expression by directly binding its 3'-untranslated region (UTR). Furthermore, miR-128-3p activation partially reversed the acceerative effect of TF on SIRT1 expression. Taken together, TF exhibits a strong nephroprotective ability to suppress CaOx-induced kidney damage through the recovery of the antioxidant defense system regulated by miR-128-3p/SIRT1 axis. These findings provide novel insights for the prevention and treatment of renal calculus.

## Introduction

Nephrolithiasis, or renal calculi, is a commonly diagnosed urological disease with a global prevalence rate of 9% in adults 1. A fast-paced lifestyle, global warming, and unhealthy or irregular diet are regarded as the critical contributors of the increasing occurrence of kidney stones worldwide 2. Despite the fact that great improvement has been achieved in minimally invasive surgical techniques, the recurrence rate of renal stones within 5 years remains nearly 50% after the first surgery 3. It is warranted to find potential therapeutic agents which have satisfactory therapeutic results and few adverse effects for kidney stone patients. Calcium oxalate (CaOx), the most common constituent of renal calculus, can cause serious intrarenal inflammation and tubular cell injury, consequently promoting more crystal deposition; the oxidative stress injury caused by excessive generation of reactive oxygen species (ROS) are involved in the germination and development of kidney stone disease 4, 5.

Theaflavin (TF) is a distinctive derivative of benzotropolones first extracted from black tea, and has been shown to have antioxidant effects for its functions of radical scavenging and metal chelation 6, 7. A number of studies have revealed the treatment efficacy of TF in various diseases, such as cardiovascular disease 8, inflammatory disorders 9, fatty liver 10, and bacterial and viral infection 11. These beneficial effects have been attributed to the regulation of endogenic antioxidant systems and scavenging of excessive ROS 12, 13. Recently, TF has been reported to improve the activities of key enzymes of carbohydrate metabolism in diabetic rats, exhibiting renal protective effects via its antioxidant activity 14. In addition, TF was reported to exhibit anti-inflammatory activity in mouse model of inflammation, by downregulating the expression of inflammatory gene COX-2, TNF-a, and iNOS, and inhibiting NF-κB signaling pathway 15. Furthermore, the inhibition of CaOx crystal induced inflammation response was also regarded as an effective strategy to ameliorate tubular cell damage and prevent kidney stone formation 16, 17. However, the treatment effect of TF on nephrolithiasis remains unclear.

Many previous reports have demonstrated that microRNAs (miRNAs) are greatly involved in tubular cell oxidative stress injury and the pathogenesis of CaOx renal stones. It was reported that miRNAs could increase CaOx-induced kidney tubular cell injury and serve as potential therapeutic targets or biomarkers for renal calculi, including miR-21 and miR-155 18, 19. Our published paper revealed that the interaction of lncRNA H19 with miR-216b contributed to the development of CaOx nephrocalcinosis-induced oxidative stress and tubular cell injury through the regulation of HMGB1/TLR4/NF-kB signaling pathway 5. Several studies also identified some miRNAs exerting protective effects in the formation of kidney stones, such as miR‑30c‑5p 20, miR-93-5p 17, miR-34a 21, and miR-142a 22. All in all, miRNAs work in a variety of roles in kidney stone disease. miR-128 was reported to regulate MAPK signaling and the generation of ROS and pro-inflammatory cytokines in different types of cells and tissues, and played a promotive role in kidney inflammation and fibrosis 23, 24.

SIRT1 is a nicotinamide adenine dinucleotide (NAD+)-dependent histone deacetylase that exerts anti-inflammatory and anti-oxidative effects on pathological process in several organs, including kidney 25, 26. In the present research, we found that TF inhibited CaOx-induced kidney oxidative stress injury and alleviated kidney crystal deposition by regulating miR-128/SIRT1 axis. Thus, our study provides new insights for the administration of nephrolithiasis.

## Materials and methods

### Animals and experimental protocol

All experimental procedures were performed following the rules of the National Institutes of Health Guide for the Care and Use of Laboratory Animals, and obtained approval from the Ethics Committee of Tongji Hospital, Huazhong University of Science and Technology. 6-8-week-old C57BL/6J male mice were used for the animal experiments, and the CaOx nephrocalcinosis mouse model was established using the methods previously described 5. In brief, mice received intraperitoneal injection with vehicle (saline) or glyoxylate (Gly; 75 mg/kg/d; 200 μl) on days 8 through 14. Mice in TF (MCE, HY-N0243) intervention groups were daily treated with TF by gavage at three different doses (20, 50, and 100 mg/kg) on days 1 through 14. Theaflavin dosages were based on two previous studies 6, 27. To discuss the effect of miR-128-3p, mice were injected with a long-lasting agonist of miR-128-3p (80 mg/kg, 200 µL; Ribo Biotech, China) on day 1 and 8 via tail vein. Dosages of long-lasting miR-128-3p agonist were based on the manufacturer's instructions and our previous published study 5. Two weeks later, all animals were sacrificed, and kidney samples were collected and fixed for further assessment.

### Detection of CaOx crystals deposition

Paraffin-embedded kidney sections (4 μm) were stained with hematoxylin-eosin (HE) following the standard procedures and then visualized by a polarized light optical microscope (Zeiss, Germany). Besides, Pizzolato staining method was used to determine CaOx crystal deposition. The percent area of crystal deposition per section was evaluated quantitatively by Image J software (National Institutes of Health, USA).

### Tubular injury and cell apoptosis

Slices of renal tissues were stained with Periodic acid-Schiff (PAS) to evaluate tubular injury. Positive cells and the percentages of injured renal tubular were calculated in 10 randomly chosen fields (×200 magnification) in each section. TUNEL staining was performed to determine renal tubular cell with a commercial kit (Roche, Switzerland).

### Immunohistochemistry (IHC)

Mouse kidney slices were first stained with haematoxylin-eosin (HE) according to standard histopathological techniques. For IHC staining, the slices were incubated with primary antibodies for SOD2 (1:100, Boster, BM4813), NOX2 (1:100, Boster, BA2811), and SIRT1 (1:100, Abcam, ab32441) overnight at 4 °C, and then visualized by an Envision HRP Polymer system. Image capture (×200 magnification) was performed with an Olympus BX51 microscope. Positive cells were analyzed with Image J software.

### Fluorescence *in situ* hybridization (FISH)

Cy3-labeled mmu-miR-128-3p nucleic acid probe was purchased from Ribo Biotech (China). Experiment was carried out using a commercial kit (Ribo Biotech) following the standard procedures. All pictures were captured with a fluorescence microscope.

### Cell culture and transfection

HK-2 cell line was purchased from American Type Culture Collection (ATCC) and maintained in DMEM medium supplemented with 10% FBS in a humidified atmosphere (5% CO_2_, 37 °C). To establish an *in vitro* model, HK-2 cells were treated with or without TF (20 and 50 μg/ml) for 2 h followed by incubation with 100 mg/ml of calcium oxalate monohydrate (COM, Sigma-Aldrich, C0350000) for 24 h. Theaflavin dosages were based on previous studies and cell viability assay (data not shown) 28-30. To upregulate miR-128-3p, the miR-128-3p mimic or negative control mimic (Ribo Biotech, China) were transfected into HK-2 cells with riboFECT CP following manufacturer's instructions.

### Measurement of LDH, H_2_O_2_, SOD, and MDA

The released concentration of LDH in the culture medium was measured using a LDH Cytotoxicity Assay Kit (Jiancheng Biotech, China). LDH levels were determined spectrophotometrically at 450 nm. The H_2_O_2_ levels in culture medium were assessed with a H_2_O_2_ kit (Jiancheng Biotech, China), results were read at 595 nm. To determine the SOD and MDA concentrations, cells receiving different treatments were homogenized and the supernatants were collected after centrifugation using commercial kits (Beyotime Biotech, China). Briefly, for the evaluation of SOD activity, 20 µl of supernatants were mixed with 160 µl of NBT reagent and 20 µl of reaction reagent and held at 37 °C for 30 min, the levels were analyzed at a 560 nm wavelength; for MDA content, 100 µl of the supernatants were mixed with 200 µl of the TBA reaction solution, and then incubated at 95 °C for 60 min, after the mixture was cooled down, the results were determined at a 532 nm wavelength.

### Measurement of intracellular ROS level

After receiving predesigned administration, the HK-2 cells were incubated with serum-free DMEM medium containing DCFH-DA for 30 min at 37 °C, then collected, and resuspended in PBS. The intracellular ROS levels were measured by flow cytometry, and the excitation and the emission splitter were set at 485 and 538 nm, respectively.

### qRT-PCR

Total RNA was extracted from harvested kidneys and cultured cells, and then reverse transcribed into cDNA using TRIzol reagent (Invitrogen, USA) and a PrimeScript RT reagent kit (TaKaRa, Japan). The qRT -PCR was conducted by a Bio-Rad CFX96 system using SYBR qPCR master mix (Yeasen, China) based on the manufacturer's protocol. β-actin was used as the control. Mature miRNA expression was quantitatively detected with an All-in-One miRNA qRT-PCR Detection Kit (GeneCopoeia, USA) and normalized by U6. Primer sequences used were as follows: human SIRT1, forward (TAG CCT TGT CAG ATA AGG AAG GA) and reverse (ACA GCT TCA CAG TCA ACT TTG T); human β-actin, forward (CAC CAT TGG CAA TGA GCG GTT C) and reverse (AGG TCT TTG CGG ATG TCC ACG T); mouse SIRT1 forward (ATG ACG CTG TGG CAG ATT GTT) and reverse (CCG CAA GGC GAG CAT AGA T); mouse β-actin, forward (CAT TGC TGA CAG GAT GCA GAA GG) and reverse (TGC TGG AAG GTG GAC AGT GAG G).

### Western blot analysis

The whole cell proteins were isolated following the manufacturer's instruction (Boster, China). Protein samples (25-30 µg/lane) were separated by 10% SDS-PAGE, and then transferred onto a PVDF membrane. The blots were probed with antibodies against SIRT1 (1:1000, CST, 8469S) and β-actin (1:1000, Boster, BM0627) followed by 1 hour incubation with HRP-conjugated secondary antibodies (1:3000, CST) at 25 °C. The membranes were visualized with a ChemiDoc XRS (Bio-Rad) instrument with ECL western blotting reagent.

### Luciferase reporter assay

The fragments of the 3'-UTR of SIRT1 containing the predicted wild-type binding site of miR-128-3p or the mutated site were amplified and introduced into psiCHECK-2 vectors (Promega, USA), named as SIRT1-WT and SIRT1-MUT. Then, HK-2 cells were cotransfected with 200 ng these constructed vectors and 20uM miR-128-3p mimic or mimic control using Lipofectamine 3000 (Invitrogen, USA). Cells were lysed and analyzed for luciferase activity with a Dual-Luciferase Reporter Assay System (Promega, USA) after 48 hours transfection.

### Statistical analysis

The experimental data were presented as the mean ± standard deviation (SD) and analyzed using GaphPad Prism 8.0. software. The differences between paired group samples were evaluated by Student's t-test. The comparisons among multi-group samples were analyzed using one-way ANOVA analysis. The value of *P* < 0.05 was regarded as significant.

## Results

### TF exhibited nephroprotective effect in mice subjected to CaOx deposition-induced oxidative stress injury

**Figure 1:**
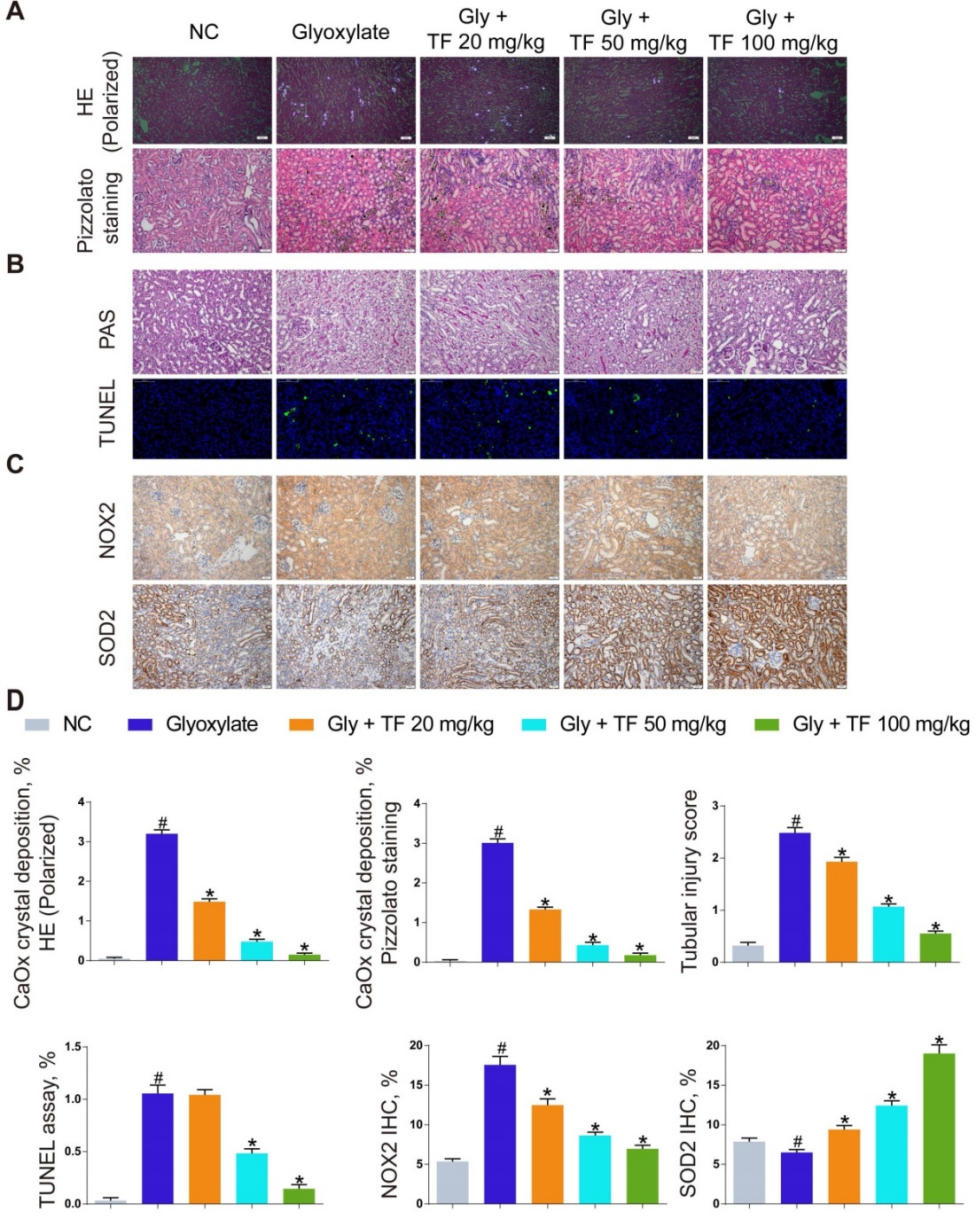
** Theaflavin alleviated CaOx nephrocalcinosis-induced oxidative stress injury and crystal deposition *in vivo*.** (A) CaOx crystal deposition in glyoxylate-induced kidney CaOx nephrocalcinosis mouse model with or without theaflavin treatment was detected by polarized light optical microscope (100× magnification; scale bar: 50 µm) and Pizzolato staining (200× magnification; scale bar: 50 µm). (B) PAS staining illustrating tubular injury (200× magnification; scale bar: 20 µm). TUNEL staining detected renal tubular epithelial cell death (200× magnification; scale bar: 100 µm). (C) IHC staining for NOX2 and SOD2 in CaOx nephrocalcinosis mouse kidney (200×; scale bar: 20 µm). (D) Quantification of CaOx crystal deposition, PAS staining, TUNEL staining, NOX2 and SOD2 IHC staining. n = 6 per group. The data are shown as the mean ± SD. #p < 0.05 vs. the normal control group, *p < 0.05 vs. the glyoxylate group.

TF has been widely used as a protective agent in multiple diseases for its anti-inflammatory and antioxidative capacities. To discuss the effect of TF supplementation on renal calculus, the CaOx nephrocalcinosis mice were fed with the low-, moderate-, and high-dose of TF, respectively. As shown in Figure 1A and 1D, a significant decrease of CaOx crystal deposition was observed in the TF treatment groups, and the declining trend was shown in a dose-dependent manner. Besides, the results in Figure 1B and 1D revealed that TF supplementation remarkably reduced the tissue damage and TUNEL-positive cells in kidneys. We further evaluated the effect of TF supplementation on the oxidative stress in mice kidneys, IHC analyses indicated stronger NOX2 positive staining and weaker SOD2 positive staining in crystal group mice compared to control group mice; TF treatment could reverse these alterations and high-dose of TF obtain the most improvement (Figure 1C and 1D). These results showed that TF treatment could alleviate CaOx nephrocalcinosis-induced oxidative stress injury and crystal deposition *in vivo*.

### TF reduced COM-induced oxidative stress injury in HK-2 cells

**Figure 2:**
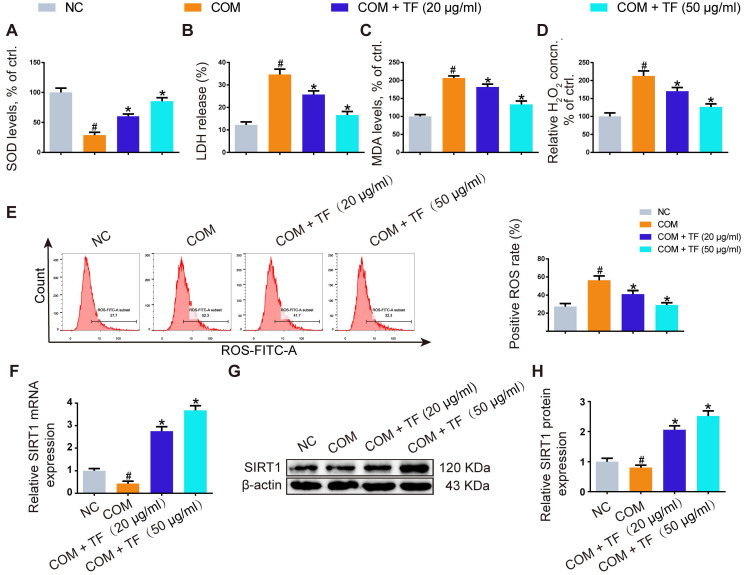
** Theaflavin inhibited COM-induced renal tubular epithelial cell oxidative stress injury *in vitro*.** SOD level (A), LDH release (B), MDA level (C), and H_2_O_2_ concentration (D) were determined in HK-2 cells incubated with COM crystals with or without theaflavin treatment. (E) Cellular ROS production in HK-2 cells was measured by flow cytometry. qRT-PCR (F) and Western blot (G, H) analyses of SIRT1 expression in HK-2 cells incubated with COM crystals with or without theaflavin treatment. β-actin was used for normalization. The data are shown as the mean ± SD of three independent experiments. #p < 0.05 vs. the normal control group, *p < 0.05 vs. the COM group.

To evaluate whether TF could attenuate COM-induced cell injury *in vitro*, we explored its effect on the oxidative stress of HK-2 cells exposed to COM crystals. As shown in Figure 2A-2D, TF treatment (20 and 50 μg/ml) significantly enhanced the activity of SOD and decreased the levels of LDH, MDA, and H_2_O_2_. DCFH-DA fluorescence was used to determine the intracellular ROS level. COM crystals exposure caused a significant increase in ROS generation in HK-2 cells. Pretreatment with TF (20 and 50 μg/ml) significantly decreased intracellular ROS generation compared with COM-induced cells (Figure 2E). To further elucidate the underlying mechanism by which TF alleviated oxidative stress injury, we determined the SIRT1 expression level in HK-2 cells. As evident in Figure 2F-2H, qRT-PCR and western blot results demonstrated that the mRNA and protein expression levels of SIRT1 were markedly suppressed by COM exposure, but TF treatment could evidently enhance its expression. Particularly, TF at 50 μg/ml had a stronger ability to upregulate SIRT1 levels than that at 20 μg/ml.

### TF decreased CaOx nephrocalcinosis-induced kidney damage by abolishing miR-128-3p-mediated SIRT1 inhibition

**Figure 3:**
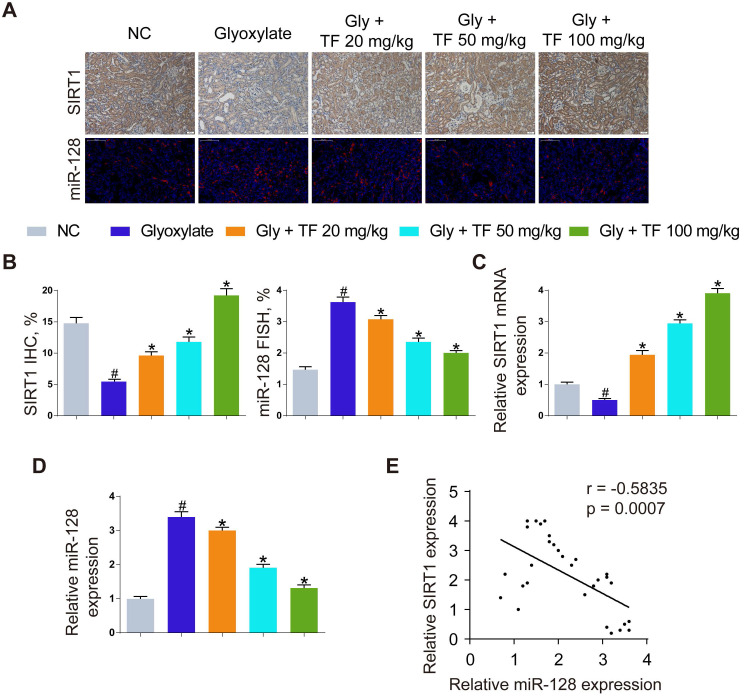
** Inhibition of oxalate calcium-induced kidney oxidative stress injury by abolishing miRNA‑128‑3p‑mediated SIRT1 inhibition.** (A) IHC was used to analyze SIRT1 expression, and FISH to detect miR-128 expression in renal tissues (200×; scale bar: 100 µm). (B) Quantification of SIRT1 IHC staining, and miR-128 FISH. (C, D) qRT-PCR was used to assess SIRT1 and miR-128 expression in kidney samples. β-actin and U6 RNA were used for normalization, respectively. (E) Pearson's correlation coefficient analysis of the expression levels of miR-128 and SIRT1 in CaOx nephrocalcinosis mouse kidney. n = 6 per group. The data are shown as the mean ± SD. #p < 0.05 vs. the normal control group, *p < 0.05 vs. the glyoxylate group.

**Figure 4:**
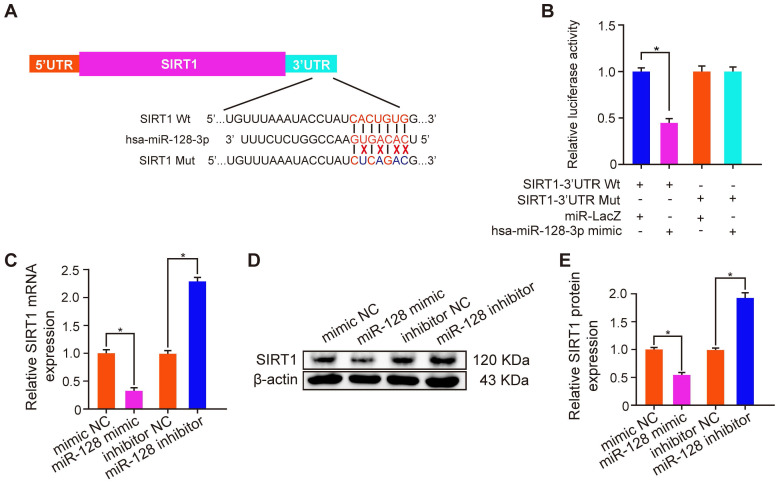
** miR-128-3p inhibited SIRT1 expression by directly binding its 3'-UTR.** (A) Schematic diagram of miR-128 targeting mutant and wild type seed sequences in the 3'-UTR of SIRT1. (B) Luciferase reporters harbouring putative target sites in the wild type and mutant 3'-UTRs of SIRT1 were cotransfected with 100 nM miR-128 mimic in HK-2 cells. qRT-PCR (C) and Western blot (D, E) were performed to detect the expression of SIRT1 in HK-2 cells transfected with the miR-128 mimic or inhibitor. β-actin served as an internal control. The data are shown as the mean ± SD of three independent experiments.

To explore the mechanism how TF increased the SIRT1 expression to exert a nephroprotective role, we detected the expression levels of SIRT1 and miR-128-3p in the harvested mice kidneys. IHC and FISH assays demonstrated decreased staining of SIRT1 and increased staining of miR-128-3p in the kidneys of crystal group mice compared to that of control group mice; otherwise, strong IHC staining of SIRT1 and weak FISH staining of miR-128-3p were observed in a TF-dependent manner (Figure 3A and 3B). We further used qRT-PCR to determine the expression levels of SIRT1 and miR-128-3p in the kidneys, and similar results were detected (Figure 3C and 3D). The result of Pearson's correlation analysis confirmed a negative relationship between SIRT1 expression and miR-128-3p expression (Figure 3E). Bioinformatics analysis indicated that the 3'-UTR of SIRT1 contained conserved, putative miR-128-3p targeting sites. A luciferase reporter system was established to verify the binding of miR-128-3p to SIRT1 mRNA (Figure 4A). Luciferase reporter vectors carrying either the wild-type (SIRT1-3'-UTR Wt) or mutated 3'-UTR of SIRT1 (SIRT1-3'-UTR Mut) were introduced into HK-2 cells. The results showed that miR-128-3p overexpression significantly inhibited the luciferase activity of cells transfected with SIRT1-3'-UTR Wt, while failed to reduce that of cells with SIRT1-3'-UTR Mut (Figure 4B). Furthermore, qRT-PCR and western blot assays found compared to NC group, miR-128-3p mimic obviously downregulated the mRNA and protein levels of SIRT1 but miR-128-3p inhibitor upregulated the levels (Figure 4C-4E). These data indicated that miR-128-3p negatively mediated the SIRT1 expression by targeting its 3'-UTR.

### miR-128-3p partially reversed the effect of TF on CaOx nephrocalcinosis-induced oxidative stress injury and crystal deposition *in vivo*

**Figure 5:**
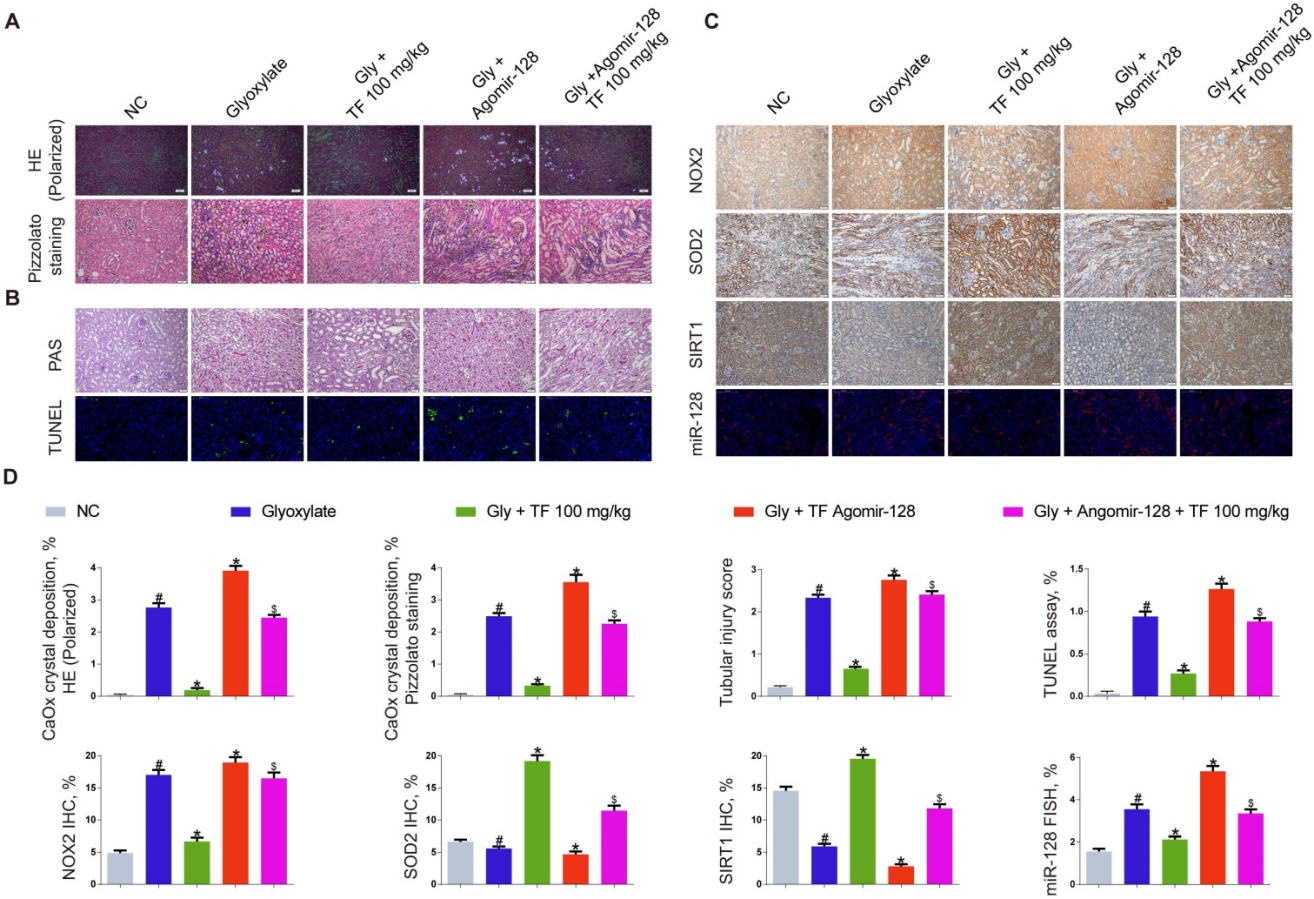
** miR-128-3p partially reversed the effect of theaflavin on CaOx nephrocalcinosis-induced oxidative stress injury and crystal deposition *in vivo*.** (A) CaOx crystal deposition in in theaflavin- and/or Agomir-128-treated mice was detected by polarized light optical microscope (100× magnification; scale bar: 50 µm) and Pizzolato staining (200× magnification; scale bar: 50 µm). (B) PAS staining illustrated tubular injury (200× magnification; scale bar: 20 µm). TUNEL staining detected renal tubular epithelial cell death (200× magnification; scale bar: 100 µm). (C) IHC was used to analyze NOX2, SOD2, and SIRT1 expression, and FISH to detect miR-128 expression in renal tissues (200×; scale bar: 100 µm). (D) Quantification of CaOx crystal deposition, PAS staining, TUNEL staining, NOX2, SOD2, and SIRT1 IHC staining, and miR-128 FISH. n = 6 per group. The data are shown as the mean ± SD. #p < 0.05 vs. the normal control group, *p < 0.05 vs. the glyoxylate group, ^$^p < 0.05 vs. the Gly + TF 100 mg/kg group.

The miR-128-3p expression was significantly increased in the kidneys of nephrocalcinosis mice injected with agomir-128-3p (Figure 5C and 5D). The decline of crystal deposition in mice kidneys induced by 100mg/kg of TF supplementation was repressed by agomir-128-3p injection (Figure 5A and 5D). The images of PAS and TUNEL staining showed that miR-128-3p overexpression promoted the kidney damage in the nephrocalcinosis mice, and partially reversed the protective effect of TF treatment (Figure 5B and 5D). The results of IHC staining observed elevated expression of NOX2 protein and weakened levels of SOD2 and SIRT1 in kidneys of TF plus agomir-128-3p group mice than that of TF treatment group mice (Figure 5C and 5D).

### miR-128-3p partially reversed the effect of TF on COM-induced renal tubular cell oxidative stress injury *in vitro*

**Figure 6:**
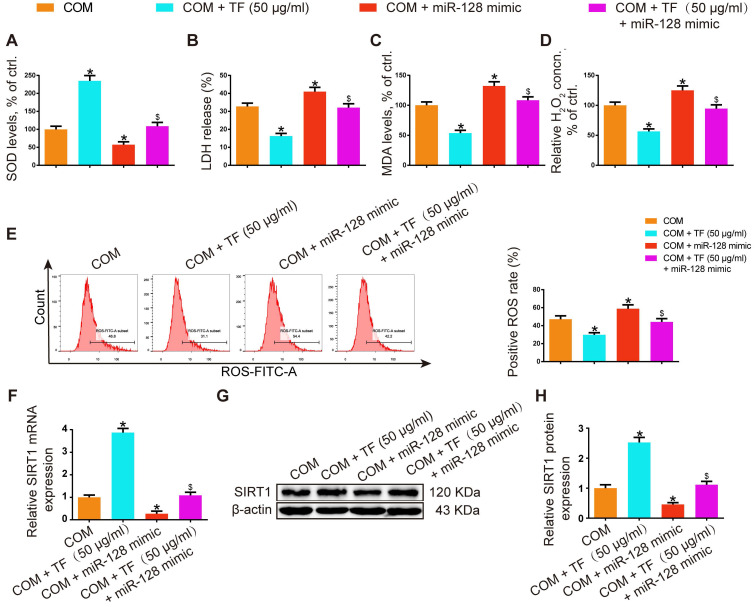
** miR-128-3p partially reversed the effect of theaflavin on COM-induced renal tubular epithelial cell oxidative stress injury *in vitro*.** SOD level (A), LDH release (B), MDA level (C), and H_2_O_2_ concentration (D) were determined in HK-2 cells treated with the miR-128 mimic and/or theaflavin. (E) Cellular ROS production in HK-2 cells was measured by flow cytometry. qRT-PCR (F) and Western blot (G, H) analyses of SIRT1 expression in HK-2 cells treated with the miR-128 mimic and/or theaflavin. β-actin was used for normalization. The data are shown as the mean ± SD of three independent experiments. *p < 0.05 vs. the COM group, ^$^p < 0.05 vs. the COM + TF (50 µg/ml) group.

In HK-2 cells, miR-128-3p overexpression showed oppositive effects against 50 μg/ml of TF treatment in COM-induced oxidative injury of HK-2 cell. The activity of SOD was dramatically suppressed in the TF + miR-128-3p mimic group, while the levels of LDH, MDA, and H_2_O_2_ were increased (Figure 6A-6D). TF treatment significantly reduced COM-induced ROS generation in HK-2 cells, whereas miR-128-3p overexpression partially reversed this effect (Figure 6E). Furthermore, qRT-PCR and WB results also demonstrated that the upregulation effect of TF on SIRT1 expression in HK-2 cells was weakened by miR-128-3p mimic transfection (Figure 6F-6H). These results suggested that the protective effect of TF on COM-induced cell oxidative injury was declined by miR-128-3p.

## Discussion

Renal calculus is a worldwide health problem, whose incidence and prevalence are increasing remarkably, placing a heavy burden on family and society. Over the past decades, the understanding about the specific mechanism of kidney stone formation remains poor. Generally, crystal nucleation, adhesive, growth, and aggregation are involved in stone formation, and crystal-cell interaction is regarded as one of the earliest processes 31. The exposure of high oxalate and/or CaOx crystals to renal tubular cells induced excessive generation of free radicals, developed oxidative stress, and eventually caused the disruption of cell membranes and renal oxidative injury 32, 33. Crystal-ROS-mediated kidney injury might promote stone formation through increasing the number of cellular debris for crystal nucleation and aggregation 34.

In the past few years, the role of oxidative stress in renal calculus formation have been discussed in many published papers. We have reported that the interaction between lncRNA H19 and miR-216b promoted tubular cell injury via ROS overproduction in the process of kidney stone formation *in vivo* and *in vitro* 5. NADPH oxidase is confirmed as the main source of free radicals in kidneys, Liang et al. found that the activation of androgen receptor (AR) signaling contributed to the upregulation of NADPH oxidase, which increased oxalate biosynthesis and oxidative stress, inducing kidney tubular injury and promoting stone formation 35. In another study, estrogen/estrogen receptor beta (ERβ) exerted protective effect on renal CaOx crystal formation by inhibiting liver oxalate biosynthesis and reducing the expression of NADPH oxidase subunit 2 (NOX2) 36. Sun et al. reported pretreatment with taurine markedly reduced oxidative stress by promoting SOD2 activity and decreasing MDA concentration, and also suppressed ROS-dependent autophagy through activating Akt/mTOR pathway, thus alleviating crystal-induced oxidative injury in kidney tissues and HK-2 cells 37. Liu et al. described the complete role of inflammatory pyroptotic in the CaOx crystal-triggered tubular cell damage, and H3 relaxin supplementation provided direct treatment for this particular form of inflammatory injury in crystalline nephropathies via the ATP-depleted protection 38. These data suggested that targeting oxidative stress injury might become an effective therapeutic management for the treatment of CaOx renal calculus. In our study, we illustrated that TF treatment could significantly ameliorate CaOx crystal-induced kidney oxidative stress injury and decrease crystal deposition in mice kidneys.

As a main bioactive compound extracted from black tea, TF has been well studied for its various functions on health care. It has been reported that TF could exert protective effect against oxidative damage on a variety of diseases via radical-scavenging 39, 40. Excessive accumulation of ROS can result in the cartilage homeostasis disruption, Li et al. found that TFs inhibited ROS overproduction in cartilage degeneration, and reduced cell apoptosis and DNA damage induced by oxidative stress via improving the activity of glutathione peroxidase 1 (GPx1) and catalase 41. Wang et al. reported that treatment with TF could attenuate the injurious effects of ethanol administration-induced oxidative stress on gastric mucosa epithelial cells by inhibiting several mitogen-activated protein kinase pathways 28. Besides, Zhang et al. revealed the neuroprotective effect of TFs against H_2_O_2_-induced apoptosis in neural cells (PC12), providing the foundations for the treatment of oxidative stress-caused neurodegenerative diseases using TFs 42. For kidney diseases, administration of TF increased the antioxidant status through suppressing the lipid peroxidation and hydroperoxides in kidney tissues, reducing the kidney damage caused by hyperglycemia 14; otherwise, Ali et al. also found TFs supplementation improved renal function in renal malfunctional rats caused by high-arginine diet 43. Consistent with these previous reports, we demonstrated a significant nephroprotective role of TF on CaOx crystal-induced tubular cell injury in animal and cell experiments, which was sustained by these findings that TF treatment obviously decreased the generation of ROS, reduced the level of MDA, LDH, and H_2_O_2_, and increased the activity of SOD.

As a member of NAD^+^-dependent deacylase, SIRT1 has been reported to correlate with inflammatory response and metabolic disorders in various tissues and organs, involved in multiple cellular signaling pathways that contribute to cytoprotective effects and metabolic regulation 44. The role of SIRT1 in kidney diseases has been focused in recent years. In streptozotocin (STZ)-induced diabetic rats, increased oxidative stress and decreased SIRT1 expression were observed in the kidneys 45. And the enhancement of SIRT1 activity was indicated to alleviate the kidney inflammation injury and oxidative damage 46-48. Besides, both our study and the study published by Hou et al. observed decreased SIRT1 expression in the renal tissues of nephrolithiasis mice established by intraperitoneal administration of glyoxalate 49. Consistently, we observed that TF reduced kidney oxidative injury via upregulating the expression of SIRT1 in crystal group mice.

Potential clinical application of miRNAs in kidney diseases is increasing and the role of miRNAs in kidney stone formation has become a hot topic. When HK-2 cells exposed to CaOx monohydrate crystals, Wang et al. found the significant downregualtion of miR-34a and upregulation of CD44, and moreover, miR-34a overexpression could suppress CD44 expression and reduce crystal-cell adhesion both *in vitro* and *in vivo* experiments 21. miRNA was also reported to be involved in the oxalate homeostasis, Anbazhagan et al. identified miR-125b as a novel regulator of SLC26A6 that was a key multifunctional anion exchanger regulating the intestine oxalate secretion, whose deletion contributed to a higher risk for nephrolithiasis 50. Based on the prediction result of TargetScan and the previous study, we first determined the expression status of miR-128-3p and SIRT1 in these experimental mice, and observed a reduction of miR-128-3p level, while an increased SIRT1 expression in the kidneys of crystal group mice that received TF supplementation. Then, the result of Pearson correlation coefficient analysis showed a negative correlation between miR-128-3p expression and SIRT1 expression. Moreover, qRT-PCR, WB, and luciferase reporter assays further confirmed that miR-128-3p directly targeted the 3'-UTR of SIRT1 to inhibit its expression. Furthermore, we found miR-128-3p activation partially reversed the accelerative effect of TF on SIRT1 expression, and reduced the nephroprotective effect of TF on CaOx crystal-induced oxidative injury *in vivo* and *in vitro*. These results indicate that TF inhibited oxalate calcium-induced kidney oxidative stress injury and crystal deposition by abolishing miR-128-3p-mediated SIRT1 inhibition.

Taken together, our present study confirmed that TF supplementation exhibited a protective effect against oxidative injury in kidney tissues and cells exposed to CaOx crystals. It was further found that TF might reduce kidney damage caused by crystal deposition via mediating the miR-128-3p/SIRT1 axis. These findings suggest miR-128-3p and SIRT1 may serve as new targets for the therapy of CaOx nephrolithiasis, and provide a potential use of TF in oxalate kidney injury, as well as kidney stone formation.
